# The olivocochlear system and protection from acoustic trauma: a mini literature review

**DOI:** 10.3389/fnsys.2015.00094

**Published:** 2015-06-22

**Authors:** Adrian Fuente

**Affiliations:** Faculté de médecine, École d’orthophonie et d’audiologie, Université de MontréalMontréal, QC, Canada

**Keywords:** noise, hearing loss, temporary threshold shift (TTS), permanent threshold shift (PTS), efferent auditory system, olivocochlear bundle, noise-induced hearing loss (NIHL)

## Abstract

Large intersubject variability in the susceptibility to noise-induced hearing loss (NIHL) is known to occur in both humans and animals. It has been suggested that the olivocochlear system (OCS) plays a significant role in protecting the cochlea from exposure to high levels of noise. A mini literature review about the scientific evidence from animal and human studies about the association between the function of the OCS and susceptibility to NIHL was carried out. Animal data consistently show that de-efferented ears exhibit larger temporary threshold shift (TTS) and permanent threshold shift (PTS) than efferented ears. Data from human studies do not consistently show a correlation between the strength of the OCS function and amount of TTS. Further research on human subjects is required to determine how the OCS function could be used to predict susceptibility to NIHL in individual subjects.

## Introduction

Noise-induced hearing loss (NIHL) is one of the most prevalent work-related health conditions around the world. Currently, in the United States alone, around 26 million people (15 percent of the population between 20 and 69 years) have hearing loss that may have been caused by exposure to noise at work or in leisure activities (National Institute on Deafness and other Communication Disorders (NIDCD), 2014). Large intersubject variability in susceptibility to NIHL is known to occur (Cody and Robertson, 1983). However, the mechanisms underlying such differences are poorly understood. It has been extensively suggested that the olivocochlear system (OCS), initially described by Rasmussen (1946, 1953), function may relate with susceptibility to NIHL. The OCS of the mammalian inner ear consists of two subdivisions, medial system (MOCS) and lateral system (LOCS). The MOCS has its origin in medially located superior olivary complex and controls cochlear function through synaptic contacts on outer hair cells (OHCs) from the ipsilateral and contralateral brainstem. The LOCS system, which originates in the lateral superior olive, is comprised of unmyelinated neurons that mainly project to the ipsilateral cochlea ending on type I auditory nerve fibers under inner hair cells (Warr and Guinan, 1979). The aim of this manuscript was to review the scientific evidence about the association between the OCS function and susceptibility to NIHL.

## Methodology

A literature search was performed using the Web of Science Database. The strategy to find suitable articles for this review was the use of a single but well inclusive term for all research studies that have investigated the OCS. The single term used was “olivocochlear”. A total of 1, 168 entries were obtained using this term within the “topic” option of the Web of Science Database. As this mini literature review aimed to investigate research studies about the association between the OCS and protection from NIHL, all entries previously obtained were examined by reading the title of the article and the key words. Articles were included if they presented with the following key words: NIHL, acoustic trauma, acoustic injury, temporary threshold shift (TTS), permanent threshold shift (PTS), susceptibility, hearing loss. A total of 109 articles were initially selected. As the search strategy was still too broad, all abstracts (*n* = 109) were reviewed in order to determine whether they were suitable for the aim of this mini review. Both animal and human studies were included in this review. After reading the abstracts of such studies, a total of 52 articles were included, and thus the full articles were accessed. Most of the articles not included in this review were eliminated because they were not exploring a possible association between the OCS and NIHL. In addition, some studies were excluded as they were reviews or conference presentations, or because the full article was not found (*n* = 2).

## Animal Studies: OCS and Protection for Acoustic Trauma

Initially, Trahiotis and Elliott (1970) did not find a significant difference in TTS between cats that had a section of the OCS and who were exposed to a broadband noise of 107 dB SPL during 10 min, and a control group of cats. However, more than 10 years later Handrock and Zeisberg (1982) demonstrated that guinea pigs with transection of the OCS presented with significantly lower and longer N1 amplitude as compared to control group animals after a noise exposure of 125 dB for 30 min. Similarly, Rajan and Johnstone (1983) reported that TTS from a high-frequency tone (10 kHz at 103 dB SPL for 1 min) can be decreased by contralateral stimulation or contralateral cochlear destruction in guinea pigs. The authors suggested that this reduction was the effect of the OCS. In a follow-up study, Rajan (1988a,b) found that electrical stimulation of the crossed OCS reduced the amount of TTS, as measured through compound action potential (CAP), after noise exposure in animals. In addition, the administration of strychnine could eliminate the reduction in TTS. Yamasoba and Dolan (1997) suggested that chronic strychnine administration into the cochlea inactivates the medial efferent fibers without changing hearing threshold and that the medial efferent fibers help to protect against PTS following noise exposure. In a follow-up study (Yamasoba and Dolan, 1998), using noise conditioning along with sectioning of the OCS, the authors concluded that although the OCS acts to attenuate NIHL, it may not be necessary for the acquired resistance to NIHL.

Liberman (1991) in a group of cats with sectioned middle-ear muscles did not find a significant association between OCS function and protection for acoustic injury. It is important to note, that in Liberman experiment, cats were binaurally exposed to a 6 kHz tone at 100 dB during 10 min. Further studies from (Rajan, 1989, 1990, 1995a,b, 1996, 2000, 2001a,b,c, 2003, 2005, 2007; Rajan and Johnstone, 1989) have then demonstrated that the OCS protective effect in animals depends on variables such as intensity and frequency of the noise, presence of hearing loss in the contralateral ear and whether the noise is presented monaurally or binaurally which leads to a differentiated pattern of stimulation of the uncrossed and crossed OC pathways.

Liberman and Gao (1995) investigated PTS between guinea pigs with an OCS that was surgically de-efferented and sham-operated animals. Animals were exposed to a narrow-band noise centered at 10 kHz for 2 h at a level of 109 or 112 dB. CAP, hair cell loss and stereocilia condition after noise exposure were investigated. Significant differences between surgically de-efferented and sham-operated animals were found only for CAP responses in those animals exposed at 112 dB. The authors concluded that the OCS may play a protective role for the extreme basal region of the cochlea. Reiter and Liberman (1995) proposed that the OCS protection relates to “slow” effects of OC activation rather than “fast” effects. The authors mentioned that the peak effect of the former is in frequency regions affected by 10-kHz exposures and when continuous OC stimulation is maintained for 1–2 min.

In the study carried out by Zheng et al. (1997a) OCS fibers in chinchillas were completely sectioned and then the animals were exposed to a 105 dB SPL broadband noise for 6 h. OHC function was explored through distortion product otoacoustic emissions (DPOAEs, 1.2–9.6 kHz) and cochlear microphonics (CM, 1–8 kHz). As a result of de-efferentation, the CM was decreased but DPOAEs were unchanged in de-efferented ears as compared with efferented control and sham-operated ears. Following noise exposure, the ears that were de-efferented showed significantly more depression for DPOAE input/output functions and greater decrement of CM amplitude. Differences between de-efferented and efferent-innervated ears were evident across all frequencies. However, OHC damage reflected by cytocochleogram was minimal in both efferented and de-efferented ears. The authors indicated that cochlear de-efferentation decreases the CM in chinchillas and increases the ear’s susceptibility to NIHL. In addition, they claimed that de-efferentation increases susceptibility at low frequencies as well as high frequencies. Similarly, in another animal study from the same group of researchers (Zheng et al., 1997b), it was found that de-efferented ears showed substantially more TTS, greater PTS and greater OHC damage as compared with efferent ears. Subsequently, Zheng et al. (2000) investigated the effect of de-efferentation of the OCS in animals exposed to impulse noise. No significant differences between efferented and de-efferented ears were observed for TTS (colliculus evoked potentials, CEP). However, 20 days after noise exposure values for CEP returned to pre noise exposure values in the efferented ears remaining significantly depressed in de-efferented ears. The amount of loss of OHC after noise exposure was not significant between efferented and de-efferented ears.

Zennaro et al. (1998) measured DPOAE with contralateral noise in order to obtain the attenuation in DPOAE amplitudes in guinea pigs. The animals were then exposed to a 2 kHz tone of 87 dB for 40 min, obtaining DPOAEs after this exposure (TTS). No association between the attenuation effect measured before noise exposure and the susceptibility to TTS was found. However, Maison and Liberman (2000) showed that the amount of suppression of OAEs was inversely correlated with the degree of hearing loss induced after noise exposure in a group of experimental animals.

Finally, it has been suggested that lateral OC fibers modulate cochlear nerve excitability protecting the cochlea from neural damage in acute acoustic injury (Darrow et al., 2007).

## Noise Conditioning Effect in Animal Studies [with or Without Sectioning the Olivocochlear Bundle (OCB)]

Brown et al. (1998) based on their study suggested that MOCS neurons show long-term plasticity in acoustic responsiveness that is dependent on their acoustic history. Thus, noise conditioning may have an effect on the strength of the OCS reflex. Patuzzi and Thompson (1991) measured the changes in neural and microphonic sensitivity in the basal turn of the guinea-pig cochlea produced by a 10 kHz, 115 dB SPL sound presented for 60 and 150 s. The drops in neural and microphonic sensitivity observed after overstimulation were highly correlated. The presentation of a non-traumatizing pure-tone to the contralateral ear (10 kHz, 80 dB SPL) during acoustic overstimulation reduced the amount of acoustic trauma. Transection of the OCS abolished the protective effect of the contralateral sound and significantly reduced the variability in the data. Canlon and Fransson (1995) investigated guinea pigs that were sound conditioned to a low-level, long-term pure tone stimulus (1 kHz, 81 dB SPL, 24 days) before exposure to a traumatic noise (1 kHz, 105 dB SPL, 72 h). Auditory brainstem response (ABR) thresholds and DPOAEs were obtained. The effect of a traumatic exposure (1 kHz, 105 dB SPL, 72 h) on a control group and a sound conditioned group (1 kHz, 81 dB SPL, 24 days) was determined. The amplitude of DPOAEs for the control group was reduced at all tested frequencies. The sound conditioned group showed increases in DPOAE amplitude with increases in the intensity of the primaries for all tested frequencies and statistically significant reductions from the pre-exposure values were not found. In addition, traumatic noise exposure affected nearly 100% of the OHCs at around 14 mm from the round window. The sound conditioned group showed a significantly less (50%) OHC loss than the control group. In another study, Canlon et al. (1999) demonstrated that after noise conditioning, the medial OC efferent terminals were protected. However, Kujawa and Liberman (1997), based on the results of their study suggested that conditioning-related protection may arise from a generalized stress response, which can be elicited by noise exposure, brain surgery, or a variety of other means. In another study, Kujawa and Liberman (1999) found that guinea pigs that were daily conditioned (6 h per day) with an octave-band noise at 85 dB SPL presented a reduction of PTS after a traumatic exposure to the same noise band at 109 dB SPL for 4 h. These results were observed for CAP and DPOAEs. In addition, the conditioning effect also enhanced the olivocochlear reflex strength, as measured through DPOAEs. However, different results were obtained by Peng et al. (2007) who observed that DPOAE amplitudes (1–3 kHz) increased after long-term noise conditioning along with a reduction in the olivocochlear reflex strength. Using a different approach, Attanasio et al. (1999) investigated the association between the OCS and the progressive threshold shift reduction when repeated exposures to the same sound were presented. A group of guinea pigs was de-efferented and then implanted with permanent electrodes for electrocochleographic measurements. Ten days after the operation the animals were exposed to an octave-band noise, centered at 4 kHz, at 85-dB SPL, for 10 consecutive days, 6 h on/18 h off. The hearing threshold was registered before and at the end of each exposure session. Complete recovery from TS in the control ear began after 4 days of exposure, whereas in the de-efferented ear hearing loss increased to day 7 (55 dB), with only a partial reduction (45 dB) beyond 10 days of exposure.

## Human Studies

Tachibana et al. (1992) demonstrated that transcutaneous electrostimulation (TE) around the ear reduced the TTS in a group of volunteers. One of the interpretations by the authors was that TE stimulated the OCS. However, a previous study (Collet et al., 1991) in human subjects exposed to noise did not find a correlation between TTS and the amount of TEOAE efferent suppression. Some years later, Scharf et al. (1994) reported a case study of a subject who underwent vestibular neurotomy for Ménière’s disease. Hearing thresholds (1–4 kHz) using the Békésy tracking method were obtained before and after a 15-minute exposure to a continuous 1 kHz tone at 90 dB SPL. TTS were similar between the operated and unoperated ear, and even a trend of less TTS in the operated ear as compared to the unoperated ear was found. However, Engdahl (1996) in a group of 8 subjects found a positive correlation between DPOAE (2–4 kHz) amplitude change after noise exposure (a third-octave band noise of 102 dB SPL centered at 2 kHz for 10 min) and the amount of contralateral suppression of DPOAE.

Veuillet et al. (2001) studied the association between the function of the OCS and recovery of hearing level after noise exposure. Thirty-six military subjects with acoustic trauma following impulse noise (shooting) were selected. All subjects included in the study developed a unilateral hearing loss in the range of 25–70 dB from 4 to 8 kHz. Pure-tone audiometry was obtained at three different times, being the first one within the first 72 h after noise exposure and then 3 and 30 days after the initial evaluation. In addition, spontaneous OAEs (SOAES) and TEOAEs with and without contralateral suppression were obtained on these three different times. There was no significant correlation between NIHL at 4, 6 and 8 kHz measured 72 h after noise exposure and the strength of the OCS function. However, a significant correlation between audiometric threshold improvement, obtained on the third evaluation session (30 days after noise exposure), and contralateral TEOAE suppression was obtained. However, Wagner et al. (2005) did not find a correlation between the amount of contralateral suppression and individual TTS in a group of human subjects. Similarly, Shupak et al. (2007) did not find an association between baseline medial OC reflex strength and pure-tone thresholds after 2 years of noise exposure in a cohort of 135 noise-exposed workers. In addition, other human studies have not found a significant correlation between DPOAE efferent suppression and shifts in pure-tone thresholds or shifts in DPOAE levels after occupational noise exposure of one workday (Müller and Janssen, 2008) or after 3 h of discotheque music (Müller et al., 2010). Similarly, Hannah et al. (2014) found that the amount of TTS was not predicted from TEOAE efferent suppression amplitudes in a group of 28 subjects who listened music with an MP3 player during 1 h, at an individually determined loud listening level.

Recently, Wolpert et al. (2014) studied a group of subjects (*n* = 40) who were exposed to a 60-min. broadband noise at 94 dB SPL. DPOAE with and without contralateral acoustic stimulation using two paradigms was obtained. One of them was through the investigation of the growth function of DPOAE (input/output function) and the other one was through a fine structure analysis of DPOAE. Hearing thresholds were obtained before and after noise exposure. TTS for the purposes of analyses was calculated as the maximum postnoise threshold change found across all frequencies in both ears. Results showed a statistically significant inverse correlation between contralateral suppression as measured through the growth function paradigm, and the amount of TTS. No significant correlation between TTS and contralateral suppression, as obtained through the fine structure analysis, was found.

Finally, an association between noise exposure and the strength of the OC function has been investigated in humans. Sliwinska-Kowalska and Kotylo (2002) have shown that workers occupationally exposed to noise presented with significant lower DPOAE efferent suppression than non-exposed control subjects. Similarly, Peng et al. (2010) found that young adults users of personal listening devices had lower although not statistically significant DPOAE efferent suppression amplitudes than non-users of personal listening devices.

## Discussion

A number of animal studies have shown that the ear can be protected from sound over-exposure by activating the OCS. However, data from human studies is equivocal in demonstrating the protective role of the OCS against noise exposure. Further research in human subjects is needed to determine how OCS function can be applied to determine susceptibility to NIHL.

A question about how the OCS may have evolved to be associated with the protection against noise trauma remains. Christopher Kirk and Smith (2003) pointed out that while sustained sources of broadband noise are found in nearly all natural acoustic environments, frequency-averaged ambient noise levels in these environments rarely exceed 70 dB SPL. In this regard, new studies have shown that the OCS may still be associated with a protective effect in the presence of “non-traumatic” sounds. Maison et al. (2013) exposed animals to an 84-dB sound during 1 week. Animals were de-efferented in various degrees. The authors found that the noise caused minimal acute threshold shift and no chronic shifts in animals with normal efferent feedback. However, in de-efferented animals, they observed a cochlear neuropathy with up to 40% loss of cochlear nerve synapses with corresponding declines in ABR responses. In addition, recent studies have also found that declines in OCS may relate with and/or precede age-related hearing loss. Zhu et al. (2007) obtained DPOAE amplitudes and contralateral suppression of DPOAEs in C57 mouse from 6 to 40 weeks of age. The authors found that the contralateral suppression of DPOAEs declines quickly and precedes peripheral age-related hearing loss. Similar results have been found by Liberman et al. (2014) who found that the loss of efferent feedback in experimental animals, who were not acoustically overexposed, accelerated age-related amplitude reduction in cochlear neural responses and increased the loss of synapses between hair cells and the terminals of cochlear nerve fibers. With this new evidence showing the protective effect of the OCS without the presence of loud sound exposure, the role of the OCS for the protection from acoustic injury should be re-defined. As pointed out by Smith and Keil (2015), the noise-protective function of the OCS might represent an evolutionary byproduct with beneficial consequences for the organism.

## Conflict of Interest Statement

The author declares that the research was conducted in the absence of any commercial or financial relationships that could be construed as a potential conflict of interest.
